# Construction of a high-density genetic map and mapping of QTLs for soybean (*Glycine max*) agronomic and seed quality traits by specific length amplified fragment sequencing

**DOI:** 10.1186/s12864-018-5035-9

**Published:** 2018-08-29

**Authors:** Yanwei Zhang, Wei Li, Yanhui Lin, Lifeng Zhang, Caijie Wang, Ran Xu

**Affiliations:** Crop Research Institute, Shandong Academy of Agricultural Sciences, Jinan, 250131 China

**Keywords:** Soybean, Genetic map, SLAF-seq, QTL, Plant height, Seed weight, Quality traits

## Abstract

**Background:**

Soybean is not only an important oil crop, but also an important source of edible protein and industrial raw material. Yield-traits and quality-traits are increasingly attracting the attention of breeders. Therefore, fine mapping the QTLs associated with yield-traits and quality-traits of soybean would be helpful for soybean breeders. In the present study, a high-density linkage map was constructed to identify the QTLs for the yield-traits and quality-traits, using specific length amplified fragment sequencing (SLAF-seq).

**Results:**

SLAF-seq was performed to screen SLAF markers with 149 F_8:11_ individuals from a cross between a semi wild soybean, ‘Huapidou’, and a cultivated soybean, ‘Qihuang26’, which generated 400.91 M paired-end reads. In total, 53,132 polymorphic SLAF markers were obtained. The genetic linkage map was constructed by 5111 SLAF markers with segregation type of aa×bb. The final map, containing 20 linkage groups (LGs), was 2909.46 cM in length with an average distance of 0.57 cM between adjacent markers. The average coverage for each SLAF marker on the map was 81.26-fold in the male parent, 45.79-fold in the female parent, and 19.84-fold average in each F_8:11_ individual. According to the high-density map, 35 QTLs for plant height (PH), 100-seeds weight (SW), oil content in seeds (Oil) and protein content in seeds (Protein) were found to be distributed on 17 chromosomes, and 14 novel QTLs were identified for the first time. The physical distance of 11 QTLs was shorter than 100 Kb, suggesting a direct opportunity to find candidate genes. Furthermore, three pairs of epistatic QTLs associated with Protein involving 6 loci on 5 chromosomes were identified. Moreover, 13, 14, 7 and 9 genes, which showed tissue-specific expression patterns, might be associated with PH, SW, Oil and Protein, respectively.

**Conclusions:**

With SLAF-sequencing, some novel QTLs and important QTLs for both yield-related and quality traits were identified based on a new, high-density linkage map. Moreover, 43 genes with tissue-specific expression patterns were regarded as potential genes in further study. Our findings might be beneficial to molecular marker-assisted breeding, and could provide detailed information for accurate QTL localization.

**Electronic supplementary material:**

The online version of this article (10.1186/s12864-018-5035-9) contains supplementary material, which is available to authorized users.

## Background

Soybean is not only an important oil crop, but also an important source of edible protein and industrial raw material [1]. Agronomic traits, such as yield, plant height (PH), lodging and seed weight (SW), have been the primary focus of breeders for many years. As the major factors of the market price of soybean, seed quality traits are increasingly attracting the attention of breeders. However, the negative correlation between yield and quality of crops makes it much difficult to select for these traits [2]. Therefore, simultaneous improvement of yield and quality has become a major problem for soybean breeders.

Molecular marker-assisted selection (MAS) might be an alternative to fit the increasing global demand for soybean products [3]. A number of QTLs underlying important agronomic traits and seed quality traits have been reported over the past decades. So far, at least 196, 265, 297 and 221 QTLs controlling PH, SW, Oil and Protein have been identified respectively (www.soybase.org), based on the different genetic backgrounds, environments and statistical methods. Furthermore, large confidence intervals around QTLs make the causative gene identification difficult.

With the development of next generation sequencing technology, several methods for single nucleotide polymorphisms (SNP) discovery, such as restriction-site associated DNA sequencing (RADseq) [4, 5], genotyping-by-sequencing (GBS) [6], specific length amplified fragment sequencing (SLAF-seq) [7] have been produced, which make it possible to obtain thousands of SNPs suitable for high-density genetic map throughout the genome. SLAF markers, which have the properties of being present in large amount, being evenly distributed and avoiding repeated sequences, has been used for genetic analysis in plants, such as sesame [8], walnut [9], rice [10], sorghum [11], wax gourd [12], grape [13] and soybean [14–17]. Since the first high-density map was constructed by SLAF-seq [7], there have been several maps reported so far. Qi et al. constructed a map, including 5308 markers with 2655.68 cM in length, using a RIL population derived from a cross between ‘Charleston’ and ‘Dongnong594’ [16]. Li et al. constructed a high-density map, using a F5:8 population of 110 RILs from a cross between ‘Luheidou2’ and ‘Nanhuizao’, which was used to identified QTLs associated the isoflavone content and fatty acid composition in soybean [15, 18]. Zhang et al. reported 20 QTLs associated with phosphorus efficiency-related traits based on a high-density map constructed by SLAF-seq [17]. Cao et al. mapped QTL associated with plant height and flowering time according the map constructed by SLAF-seq using a population of 236 RILs derived from a cross between two summer planting varieties, ‘ZXD’ and ‘NN1138–2’ [14]. Nevertheless, based on the high-density map, little QTLs related to seed weight and/or protein have been reported. Therefore, we reported a high-density genetic linkage map using the SLAF-seq approach, which was based on an F_8:11_ RIL population with 149 individuals. Moreover, the QTLs associated with plant height, seed weight, oil and protein content were located and analyzed. The results presented here will aid molecular marker-assisted breeding and provide detailed information for accurate QTL localization.

## Results

### Analysis of SLAF-seq and SLAF markers

**Fig. 1:**
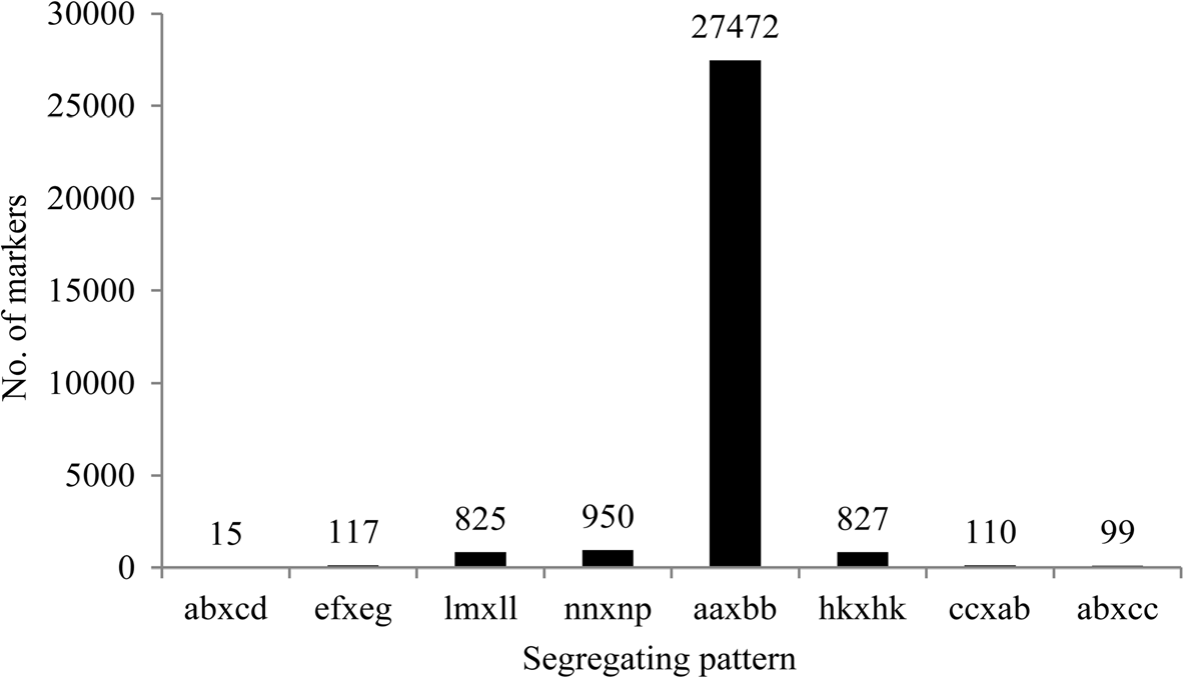
Number of markers for eight segregation patterns

DNA sequencing generated about 400.91 M pair-end reads. The Q30 (indicating a 0.1% chance of error) was 90.69% and guanine-cytosine (GC) content was 40.3%. The numbers of SLAFs in the female and male parents were 312,740 and 275,046, respectively. The numbers of SLAFs in each individual ranged from 167,933 to 237,666, with an average of 207,105. Among the 391,476 SLAF markers detected, 53,132 markers were polymorphic. All polymorphic SLAFs were then genotyped separately for all individuals. After discarding the SLAF markers lacking parent information, 30,415 markers were genotyped successfully and were classified into eight segregation types (Fig. 1). As the population was derived from a cross between two fully homozygous parents, only 27,472 markers with aa×bb type might be suitable for map construction. After filtering low-quality SLAF markers, segregation distortion markers and makers with the MLOD value ≤3, 5111 markers were used for the map construction (Additional files 1, 2, and 3). The average depth of the markers was 81.26-fold in the female parent, 45.79-fold in the male parent, and 19.84-fold in the offspring.

### The basic characteristics of the genetic map

**Fig. 2:**
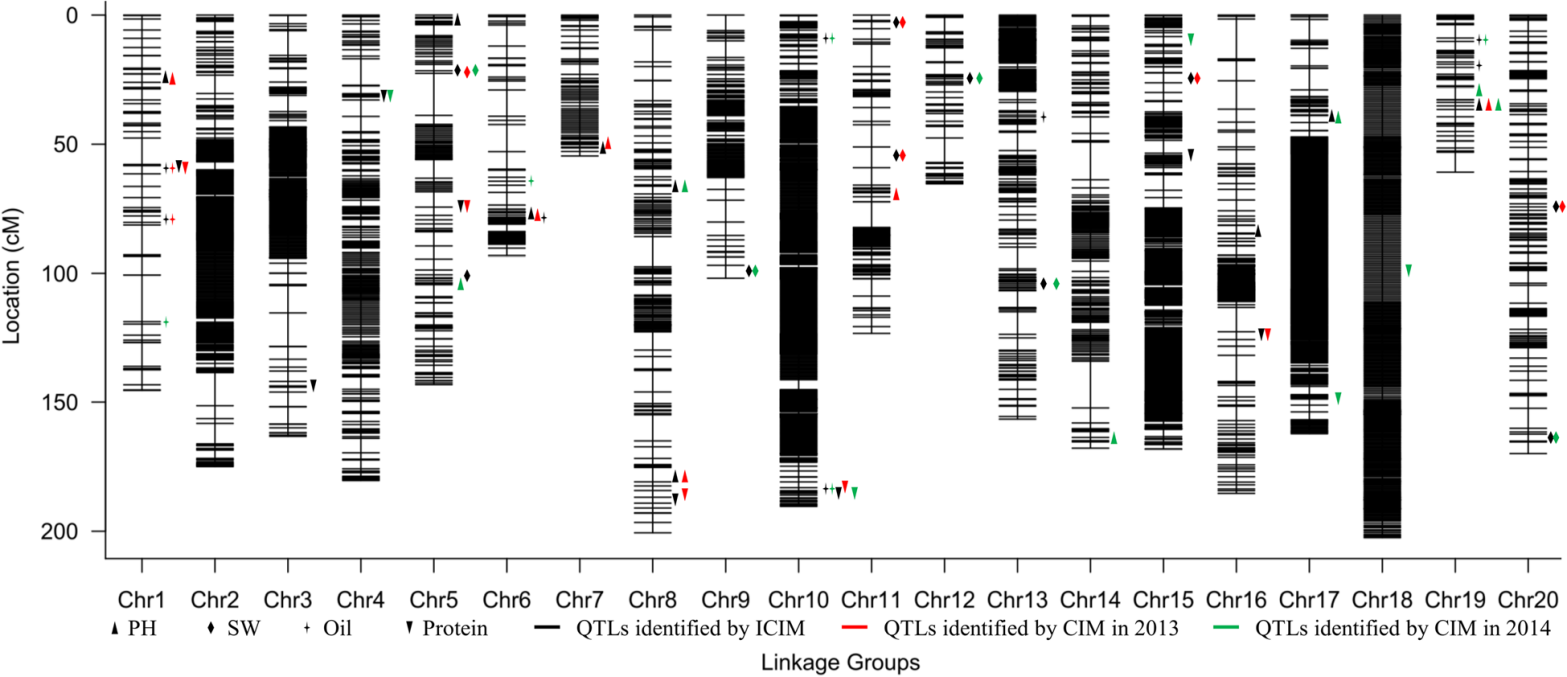
The positions of QTLs for four traits. QTLs for four traits are depicted in different shapes on the right side of each linkage group. 35 QTLs identified by ICIM are colored in black; 18 QTLs identified in 2013 by CIM are colored in red; 21 QTLs identified in 2014 by CIM are colored in green

The length of final map was 2909.46 cM, with an average distance of 0.57 cM between adjacent markers (Table 1; Fig. 2). There were 8597 SNP loci among the 5111 markers on the map. For each chromosome, the average distance ranged from 0.24 cM to 2.55 cM (Table 1). The largest linkage group was LG18 (chr18) with 480 markers, a length of 202.52 cM, and an average distance of only 0.42 cM between adjacent markers. The smallest linkage group was LG7 (chr7) with 63 markers, a length of 54.57 cM, and an average distance of 0.87 cM between adjacent markers.

**Table 1:** Description on basic characteristics of the 20 linkage groups

Linkage group ID	SLAF number	SNP number	Total length (cM)	Average distance between markers (cM)
chr1	57	87	145.47	2.55
chr2	408	628	174.9	0.43
chr3	329	523	163.13	0.5
chr4	233	373	180.32	0.77
chr5	126	174	143.23	1.14
chr6	75	107	93.21	1.24
chr7	63	110	54.57	0.87
chr8	143	216	200.62	1.4
chr9	136	219	101.89	0.75
chr10	809	1431	190.37	0.24
chr11	103	149	123.31	1.2
chr12	63	84	65.39	1.04
chr13	350	656	156.62	0.45
chr14	170	261	167.73	0.99
chr15	582	1089	168.07	0.29
chr16	239	397	185.31	0.78
chr17	568	955	162.12	0.29
chr18	480	848	202.52	0.42
chr19	48	78	60.85	1.27
chr20	129	212	169.83	1.32
total	5111	8597	2909.46	0.57

### Phenotypic evaluation

**Fig. 3:**
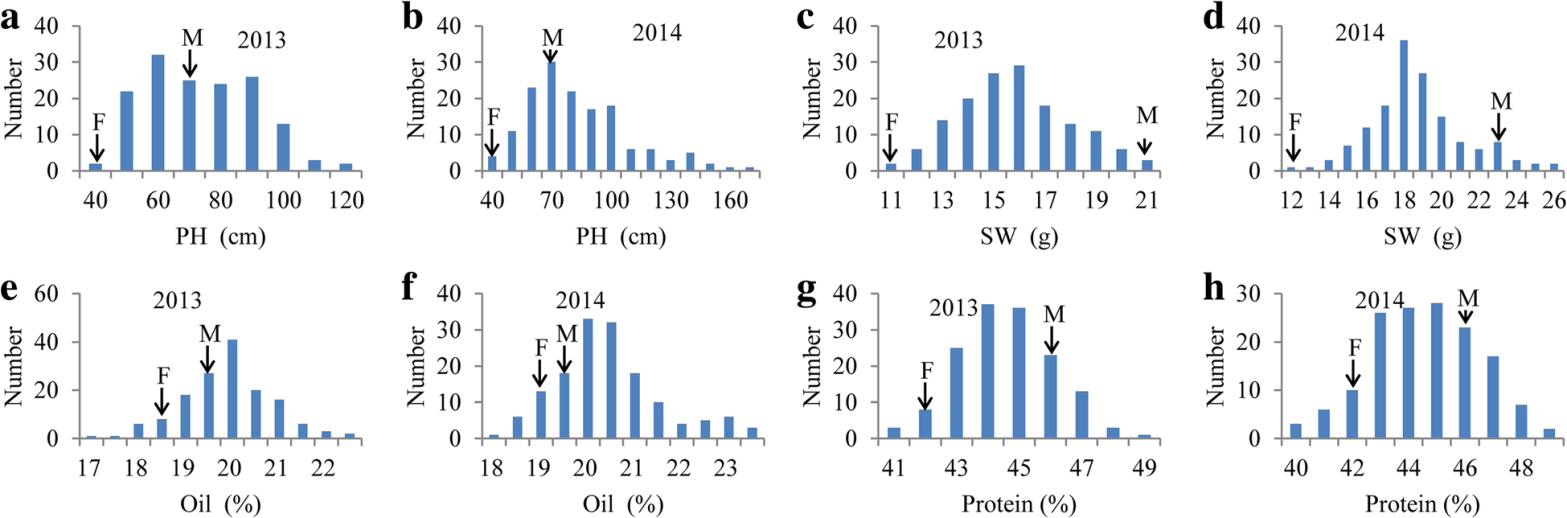
The frequency distribution for soybean traits of the RIL population and parents. F: female parent, ‘Huapidou’; M: male parent, ‘Qihuang26’; (**a**): the frequency distribution for PH in 2013; (**b**): the frequency distribution for PH in 2014; (**c**): the frequency distribution for SW in 2013; (**d**): the frequency distribution for SW in 2014; (**e**): the frequency distribution for Oil in 2013; (**f**): the frequency distribution for Oil in 2014; (**g**): the frequency distribution for Protein in 2013; (**h**): the frequency distribution for Protein in 2014

‘Huapidou’ and ‘Qihuang26’ showed a significant difference in PH, SW and Protein, but did not differ from each other in Oil significantly (Table 2; Fig. 3). However, the phenotypic values were all in a condition of continuous distribution approximately (Fig. 3). The coefficients of variation for four traits were about 20%. The heritabilities of four traits ranged from 49.58 to 82.73%. However, the heritability of Protein was only 49.58%, indicating that other factors affected Protein should be considered.

**Table 2:** Summary of soybean traits in the RIL population and parents

Trait	Year	Parents		RILs							
		Huapidou	Qihuang26	max	min	mean	SD	Skewness	Kurtosis	CV(%)	*h*^*2*^ (%)
PH (cm)	2013	73.60	42.50	126.60	46.20	79.14	17.62	0.37	−0.75	21.92	82.73
	2014	70.50	46.40	173.5	42.40	88.43	26.91	0.91	0.55	20.53	
SW (g)	2013	11.40	21.40	21.72	11.05	16.30	2.12	0.29	− 0.16	19.86	66.37
	2014	12.10	23.80	26.40	12.35	19.29	2.48	0.61	0.66	17.65	
Oil (%)	2013	18.83	19.65	22.73	17.00	20.23	0.91	−0.20	0.89	15.88	70.71
	2014	19.44	19.72	23.26	17.82	20.05	1.08	0.86	1.12	19.85	
Protein (%)	2013	42.32	46.46	49.32	41.98	45.17	1.40	0.25	−0.06	19.07	49.58
	2014	42.59	46.64	49.31	40.94	45.15	1.70	0.04	−0.39	20.31

### Analyses of additive QTLs

**Fig. 4:**
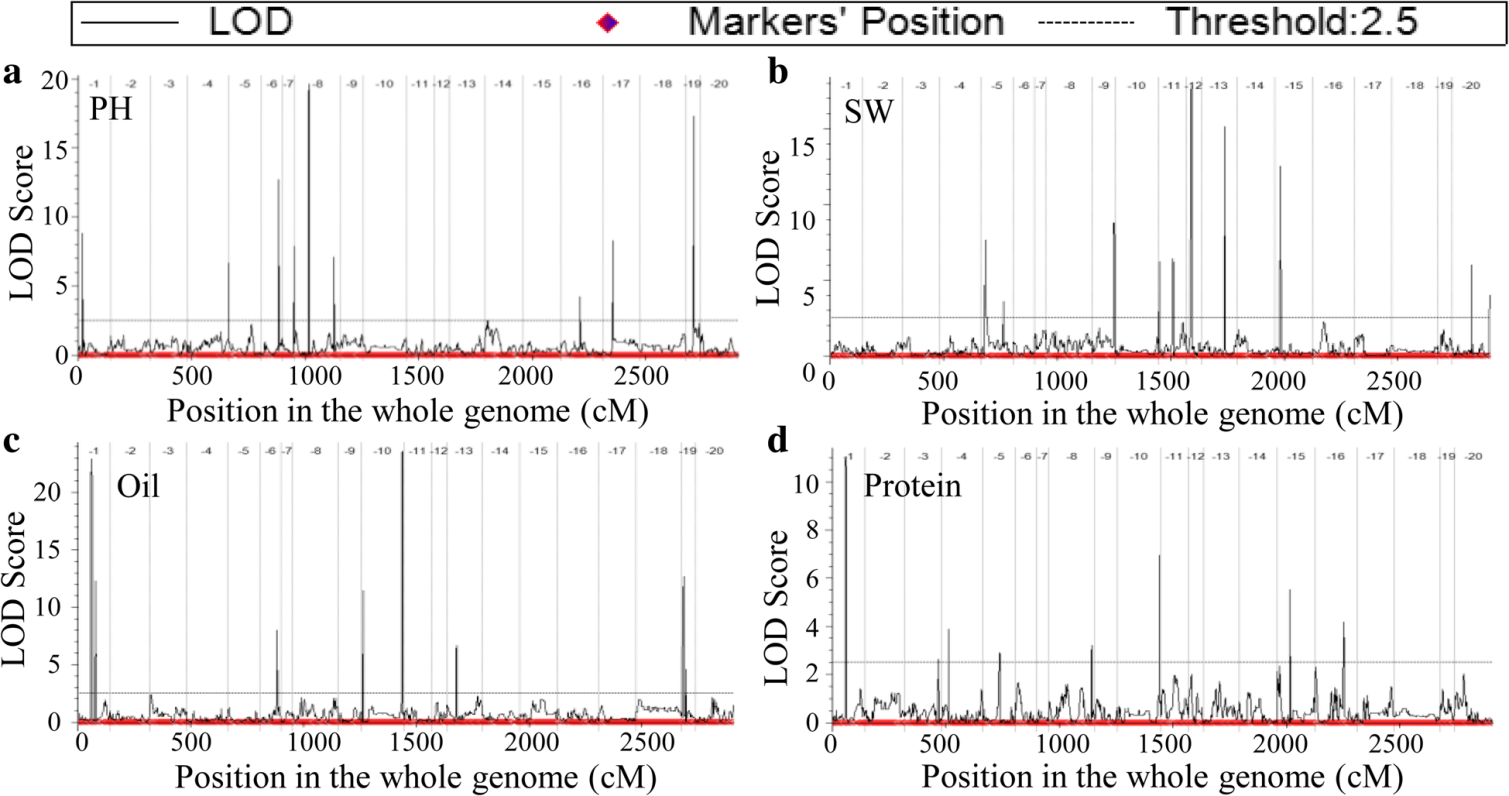
QTL mapping for the soybean traits with ICIM-ADD method. (**a**): LOD curve on the genome for PH; (**b**): LOD curve on the genome for SW; (**c**): LOD curve on the genome for Oil; (**d**): LOD curve on the genome for Protein

In total, 35 additive QTLs for PH, SW, Oil and Protein were identified on 17 chromosomes by ICIM method (Table 3; Figs. 2, 4). A single QTL explained 2.66% (*qPH16–1*) to 37.61% (*qPH8–1*) of phenotypic variance. Among the QTLs, 14 QTLs of them were observed for the first time (Table 3). A total of 21 QTLs were related to the region of the QTLs reported previously, and 19 of them were co-located in the regions with shorter intervals than previously reported, which might provide more detailed information for gene identification.

**Table 3:** QTLs identified by ICIM

QTL	Chr^a^	Left marker	Right marker	Genetic position (cM)			Physical position (bp)			LOD^b^	PVE(%)^c^	ADD^d^	QTLs reported^e^
				Start	End	Distance	Start	End	Distance				
*qPH1–1*	1	Marker4368622	Marker4217409	20.62	21.09	0.47	2,481,199	2,518,043	36,844	8.81	4.66	2.65	*qPH-1-1* [14]
*qPH5–1*	5	Marker1701487	Marker1739184	0.81	1.29	0.48	3,041,062	3,116,230	75,168	6.65	9.30	5.05	*qPH05–1* [26]
*qPH6–1*	6	Marker131534	Marker172599	76.69	77.50	0.81	17,471,671	18,171,743	700,072	12.68	7.62	5.02	*plant height 17–6* [25]
*qPH7–1*	7	Marker5882404	Marker5762234	52.83	54.57	1.74	42,930,499	42,952,373	21,874	7.86	4.09	−3.21	
*qPH8–1*	8	Marker2999607	Marker2981023	63.87	65.77	1.90	13,832,458	14,205,949	373,491	19.61	37.61	8.58	
*qPH8–2*	8	Marker2962002	Marker2806136	174.90	175.24	0.34	45,424,861	45,433,165	8304	7.09	3.94	3.65	
*qPH16–1*	16	Marker3112758	Marker3182372	81.56	84.52	2.96	6,932,336	7,381,056	448,720	4.19	2.66	−3.20	
*qPH17–1*	17	Marker403730	Marker599785	41.57	44.66	3.08	15,333,360	15,937,896	604,536	8.28	11.88	5.78	*qPH17–1* [26]
*qPH19–1*	19	Marker2154660	Marker1993462	33.77	35.14	1.36	38,782,154	38,837,787	55,633	17.29	11.56	6.43	*plant height 6–4* [19]
*qSW5–1*	5	Marker1790368	Marker1713283	21.62	22.63	1.01	5,022,424	5,076,531	54,107	7.63	7.44	0.53	*seed weight 25–1* [37]; *seedweight 50–17* [67]
*qSW5–2*	5	Marker1954345	Marker1983847	100.58	101.74	1.16	37,080,533	37,099,610	19,077	3.56	3.48	−0.15	
*qSW9–1*	9	Marker4666534	Marker4706727	96.89	101.89	5.00	13,567,369	13,932,346	364,977	8.78	9.53	0.53	*seed weight 15–6* [28]
*qSW11–1*	11	Marker1480158	Marker1628838	2.55	5.27	2.72	1,243,413	1,813,344	569,931	6.20	5.17	0.38	
*qSW11–2*	11	Marker1573686	Marker1603890	59.13	65.87	6.74	10,726,154	25,136,002	14,409,848	6.41	5.43	−0.37	*seed weight 32–1* [68]
*qSW12–1*	12	Marker5558494	Marker5592791	18.47	23.12	4.65	3,998,395	4,965,904	967,509	17.83	22.77	−0.77	
*qSW13–1*	13	Marker3613921	Marker3643913	103.37	104.05	0.68	29,371,757	29,486,787	115,030	15.12	18.55	−0.70	*seed weight 6–6* [27]; *seed weight 15–3* [28]; *seed weight* 49–14 [30]; *seed weight 44–2* [29]
*qSW15–1*	15	Marker2580154	Marker2712589	22.49	26.30	3.81	13,761,334	13,880,441	119,107	12.51	12.21	−0.46	*seed weight 2–3* [31]; *seed weight 34–12* [32]
*qSW20–1*	20	Marker809331	Marker998443	87.30	89.41	2.11	35,452,608	35,720,703	268,095	5.97	5.26	0.39	*seed weight 36–5* [32]
*qSW20–2*	20	Marker846233	Marker994705	165.31	169.83	4.52	45,972,286	46,673,050	700,764	3.96	3.97	0.35	*seed weight 50–16* [67]
*qOil1–1*	1	Marker4303320	Marker4156660	61.50	66.40	4.90	35,010,738	37,973,804	2,963,066	22.89	30.25	0.42	
*qOil1–2*	1	Marker4417270	Marker4449632	77.85	80.37	2.51	35,669,867	36,135,498	465,631	12.29	14.19	−0.24	
*qOil6–1*	6	Marker172599	Marker293115	77.50	78.32	0.82	18,171,372	18,533,013	361,641	7.98	5.59	−0.22	*seed oil 23–1* [28]
*qOil10–1*	10	Marker1115605	Marker1053573	9.00	9.81	0.82	5,243,953	5,694,905	450,952	11.41	8.12	−0.23	
*qOil10–2*	10	Marker1281634	Marker1235885	180.85	183.08	2.23	41,520,254	41,545,664	25,410	23.61	20.17	0.37	
*qOil13–1*	13	Marker3440932	Marker3635368	42.22	44.89	2.67	14,747,494	16,600,118	1,852,624	6.62	4.31	−0.09	*seed oil 24–4* [33]
*qOil19–1*	19	Marker2188637	Marker2054649	8.92	10.22	1.31	34,198,684	34,698,502	499,818	12.69	8.92	−0.24	*mqseed oil-022* [34]
*qOil19–2*	19	Marker2061159	Marker2270561	18.20	19.09	0.88	36,014,695	36,059,931	45,236	4.58	2.91	0.11	*seed oil 2–7* [35]
*qProtein1–1*	1	Marker4337527	Marker4303320	58.32	61.50	3.18	14,274,646	37,973,804	23,699,158	11.05	17.68	−0.37	
*qProtein3–1*	3	Marker6774045	Marker6819354	146.11	151.72	5.61	37,481,860	38,933,728	1,451,868	2.61	3.57	−0.14	*seed protein 21–9* [25]
*qProtein4–1*	4	Marker6548332	Marker6730760	31.65	32.83	1.18	5,276,137	5,604,533	328,396	3.86	9.85	0.23	*seed protein 19–1* [69]
*qProtein5–1*	5	Marker1695301	Marker1847746	74.43	77.65	3.23	32,108,882	32,889,638	780,756	2.90	4.05	−0.17	
*qProtein8–1*	8	Marker2934032	Marker2871125	186.80	189.12	2.32	43,600,623	43,628,816	28,193	3.19	4.56	0.18	*seed protein 21–1* [25]
*qProtein10–1*	10	Marker1235885	Marker1249450	183.08	184.24	1.16	41,545,293	42,037,748	492,455	6.94	16.83	−0.36	*seed protein 27–5* [37]; *qPro10a* [38]
*qProtein15–1*	15	Marker2482668	Marker2379482	56.98	57.12	0.13	15,668,567	15,751,338	82,771	5.51	14.36	−0.25	*seed protein 4–13* [36]
*qProtein16–1*	16	Marker3134296	Marker3275652	122.68	125.74	3.06	31,042,213	31,372,812	330,599	4.18	6.24	0.23	

^a^chr, chromosome; ^b^ LOD, logarithm of odds; ^c^ PVE, phenotypic variance explained; ^d^ ADD, additive effect; ^e^ QTL names based on soybase.org and previous reports

CIM method was also used to identify QTLs separately for 2 years. In total, 18 QTLs were observed on 11 chromosomes in 2013, and 21 QTLs were found on 15 chromosomes in 2014 (Additional file 4; Fig. 2). Of these QTLs, 31 QTLs were both identified by two methodsMoreover, *q2013PH19–1* and *q2014PH19–2* with shorter intervals than previously reported [19]*,* which were located in the same confidence intervals, might be stable across both years. It was noteworthy that *q2013Oil1–1* was placed in the same confidence intervals as *q2013Protein1–1*, and *q2014Oil10–2* was placed in the same confidence intervals as *q2013protein10–1*, which might be useful in the coordinated improvement of seed quality for soybean breeding.

### Analyses of epistatic effects

A total of 3 pairs of epistatic QTLs involving 6 loci on 5 chromosomes were identified for Protein (Table 4). The epistatic effect explained 5.49%, 4.49% and 4.06% of the PV, respectively. Pair one was composed of 2 QTLs, *qProtein2–1* and *qProtein3–2*, with the PVE of 5.49%. *qProtein5–2* was observed to have the epistatic effect with *qProtein12–1*. Meanwhile, *qProtein3–3* showed epistatic interaction with *qProtein17–1*. However, no epistatic effect was observed for PH, SW and Oil.

**Table 4:** Epistatic QTLs detected by ICIM-EPI

Trait	QTL	Chr^a^	Marker intervial	QTL	Chr^a^	Marker intervial	LOD^b^	PVE(%)^c^	Add^d^
Protein	*qProtein2–1*	2	Marker6076626 – Marker6311989	*qProtein3–2*	3	Marker6826333 – Marker6848060	8.43	5.49	−0.31
	*qProtein5–2*	5	Marker1775833 – Marker1882558	*qProtein12–1*	12	Marker5423318 – Marker5486308	7.33	4.49	−0.29
	*qProtein3–3*	3	Marker6856695 – Marker7017453	*qProtein17–1*	17	Marker379161 – Marker488109	6.73	4.06	0.24

^a^chr, chromosome; ^b^ LOD, logarithm of odds; ^c^ PVE, phenotypic variance explained; ^d^ ADD, additive effect

### Prediction of candidate genes

**Fig. 5:**
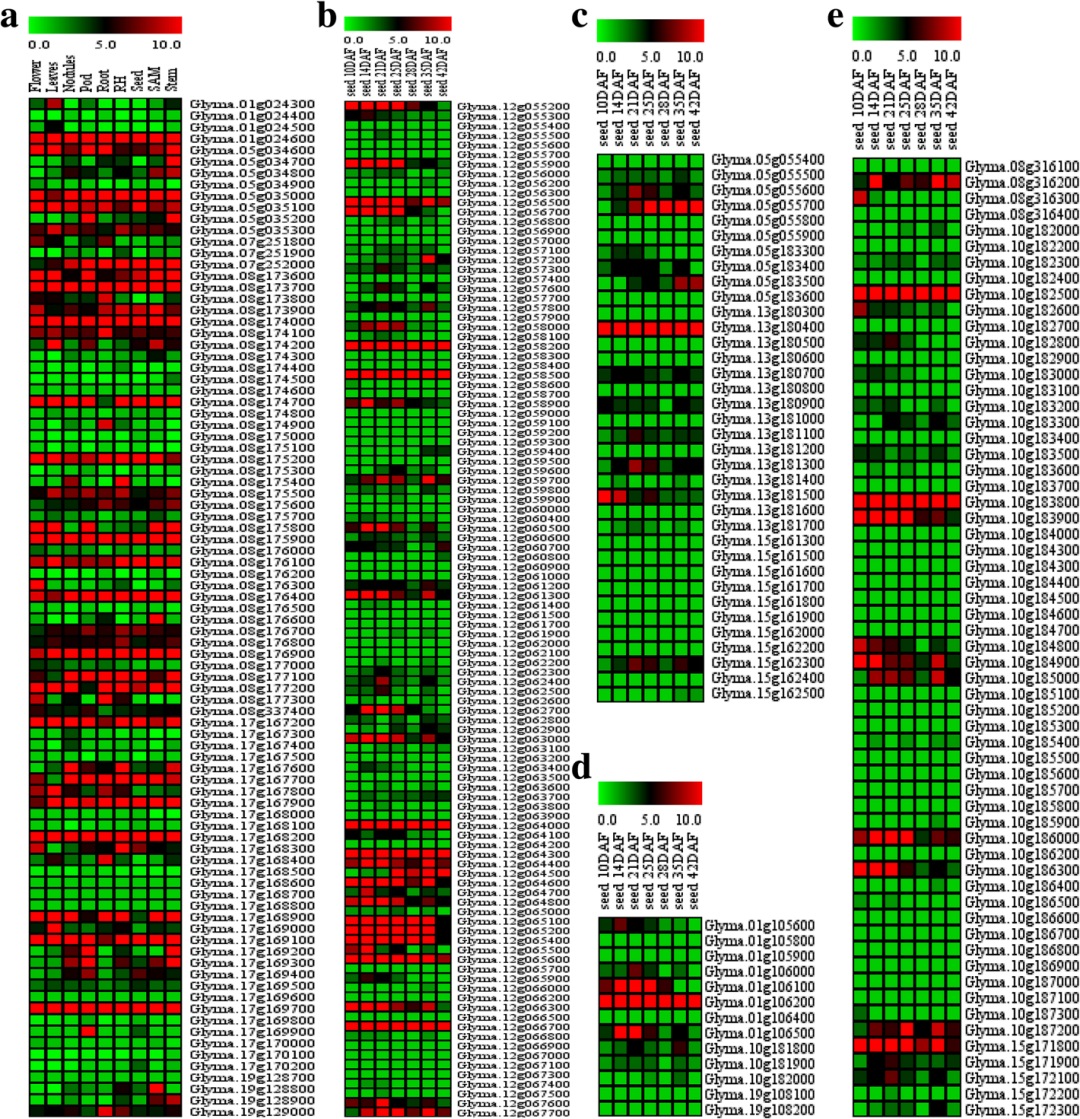
Tissue specific expression of candidate genes. RH, root hair; SAM, shoot apical meristem; DAF, day after flowering; (**a**), expression of candidate gene related to PH; (**b**), expression of candidate genes related to SW on chr12; (**c**), expression of candidate genes related to SW on chr5, chr13 and chr15; (**d**), expression of candidate genes related to Oil; (**e**), expression of candidate genes related to Protein

After filtering QTLs by the PVE and physical distance, 18 QTLs were used to mine candidate genes. According to the physical map, a total of 89, 144, 16 and 64 genes were screened in the interval of the filtered QTLs associated with PH, SW, Oil and Protein, respectively. Based on the expression data of candidate genes from phytozome and soybase (Fig. 5), 43 genes were considered to be potential candidates (Table 5). All genes from specific QTLs intervals were evaluated based on their expression pattern in different organs. In the case of genes from the QTLs associated with PH, 13 genes showed higher expression in stem and shoot apical meristem, indicating it might be considered as candidate genes related to PH (Fig. 5a). A total of 14 genes in the interval of the QTLs associated with SW, expressed in seed development stages (10 to 42 days after flowering), might participate in the pathways affecting SW (Fig. 5b and 5c). As the accumulation of oil and protein in soybean seed was throughout the seed development stage [20, 21], gene expressed sustainably in seed development stage might affect the biological process associated with oil and protein. In the present study, there were 7 genes found in the region of QTLs related to Oil, which expressed stably throughout the seed development stage, suggesting it might be associated with Oil (Fig. 5d). Meanwhile, 9 genes in the interval of QTLs for Protein, showed expression in the seed development stage, might be associated with Protein (Fig. 5e).

**Table 5:** Annotations of candidate genes

QTL	Gene	Start	Stop	Annotation
*qPH5–1*	*Glyma.05 g035000*	3,078,012	3,084,940	Xanthine/uracil permease family protein
*qPH8–1*	*Glyma.08 g173700*	13,838,458	13,841,300	Photosystem II subunit R
	*Glyma.08 g174000*	13,887,781	13,892,150	cAMP-regulated phosphoprotein 19-related protein
	*Glyma.08 g175200*	13,981,187	13,982,765	Glutathione S-transferase TAU 19
	*Glyma.08 g175800*	14,037,039	14,042,017	Aldolase-type TIM barrel family protein
	*Glyma.08 g175900*	14,046,314	14,050,528	TCP-1/cpn60 chaperonin family protein
	*Glyma.08 g177100*	14,195,428	14,196,376	
	*Glyma.08 g177200*	14,199,862	14,201,145	Arabinogalactan protein 1
*qPH8–2*	*Glyma.08 g337400*	45,423,999	45,433,706	Transducin/WD40 repeat-like superfamily protein
*qPH17–1*	*Glyma.17 g167700*	15,479,880	15,481,925	CYCLIN D3;2
	*Glyma.17 g169100*	15,650,607	15,655,215	2OG-Fe(II) oxygenase superfamily protein
	*Glyma.17 g169200*	15,679,753	15,681,009	Protein of unknown function (DUF579)
	*Glyma.17 g169700*	15,823,866	15,827,109	Sumo conjugation enzyme 1
*qSW5–1*	*Glyma.05 g055700*	5,050,728	5,055,325	Beta vacuolar processing enzyme
*qSW12–1*	*Glyma.12 g055200*	3,998,947	3,999,707	Histone superfamily protein
	*Glyma.12 g056700*	4,123,137	4,123,683	
	*Glyma.12 g058200*	4,235,968	4,237,273	HSP20-like chaperones superfamily protein
	*Glyma.12 g058500*	4,254,166	4,256,321	Adenine nucleotide alpha hydrolases-like superfamily protein
	*Glyma.12 g064000*	4,707,197	4,709,987	Heat shock protein 70
	*Glyma.12 g064300*	4,739,216	4,740,496	Pathogenesis-related thaumatin superfamily protein
	*Glyma.12 g064500*	4,752,366	4,753,273	Dessication-induced 1VOC superfamily protein
	*Glyma.12 g065100*	4,793,422	4,794,276	Histone superfamily protein
	*Glyma.12 g065200*	4,801,083	4,802,209	Histone superfamily protein
	*Glyma.12 g065400*	4,806,242	4,807,054	Histone superfamily protein
	*Glyma.12 g065600*	4,818,489	4,822,602	Ras-related small GTP-binding family protein
	*Glyma.12 g066700*	4,824,810	4,828,762	Ribosomal protein L23AB
*qSW13–1*	*Glyma.13 g180400*	29,380,779	29,383,908	Thioredoxin family protein
*qOil1–2*	*Glyma.01 g105600*	35,687,686	35,690,416	
	*Glyma.01 g106000*	35,856,497	35,858,671	Glutathione S-transferase TAU 8
	*Glyma.01 g106100*	35,881,007	35,883,036	Glutathione S-transferase TAU 8
	*Glyma.01 g106200*	35,918,034	35,919,778	Adenine nucleotide alpha hydrolases-like superfamily protein
	*Glyma.01 g106500*	36,103,010	36,105,425	
*qOil10–2*	*Glyma.10 g181800*	41,516,211	41,528,077	Calcium-binding EF-hand family protein
	*Glyma.10 g181900*	41,531,674	41,535,236	Trigalactosyldiacylglycerol 1
*qProtein8–1*	*Glyma.08 g316200*	43,611,374	43,617,841	Endoribonuclease L-PSP family protein
*qProtein10–1*	*Glyma.10 g182500*	41,577,972	41,579,422	Ribosomal protein L14
	*Glyma.10 g183800*	41,699,751	41,702,224	Differentiation and greening-like 1
	*Glyma.10 g183900*	41,719,265	41,728,269	Peptide transporter 3
	*Glyma.10 g184900*	41,781,111	41,786,726	Ureidoglycolate amidohydrolase
	*Glyma.10 g186000*	41,910,760	41,912,094	Phosphoenolpyruvate carboxylase kinase 1
	*Glyma.10 g186300*	41,926,187	41,928,530	Small nuclear ribonucleoprotein family protein
	*Glyma.10 g187200*	42,035,252	42,037,969	P-loop containing nucleoside triphosphate hydrolases superfamily protein
*qProtein15–1*	*Glyma.15 g171800*	15,667,788	15,671,786	Eukaryotic translation initiation factor 4A1

## Discussion

### Construction of a high-density genetic map based on SLAF markers

QTL mapping has been used as an efficient approach to analyze quantitative traits in plants. Parental genetic diversity and marker density are the major factors affecting the efficiency and accuracy of QTL mapping. In this study, the female parent, ‘Huapidou’, was a semi-wild soybean gerplasm, which showed high resistance to whitefly [22]. ‘Qihuang26’, with more than 46% of protein conten in seeds, was a main variety in Huang-Huai-Hai region of China. In the present study, four traits of the RIL population derived from Huapidou and Qihuang26 showed to be continuous with normal or skew normal distributions. Increasing marker density could improve the resolution of genetic map for a given mapping population [23]. SLAF-seq is an effective sequencing-based method for large-scale marker discovery and genotyping, which has been used for genetic analysis in many species [8–13, 24]. In the present study, we used 5111 high-quality SLAF markers to construct a high-density map, and a total of 8597 SNP loci were integrated into 20 LGs ultimately. This high-density genetic map, making QTL mapping more accurate and reliable, would be beneficial to MAS breeding.

### QTL mapping in soybean using a high-density map

Soybean is a primary source of plant oil and protein for humans due to its high nutritional value. PH and SW were main yield-related traits in soybean. So far, markers associated with the QTL underlying PH, SW, Oil and Protein have been mapped onto all linkage groups. In total, there were 35 QTLs for PH, SW, Oil and Protein observed using a high-density map based on an F8:11 RIL population with 149 individuals from the cross between ‘Huapidou’ and ‘Qihuang26’. Furthermore, there were 14 novel QTLs related to PH, SW, Oil and Protein, indicating the distinct genetic architecture in the population derived from cultivated soybean and semi-wild soybean. Among the novel QTLs, *qPH8–1* had the highest PVE value and the highest LOD value might be the major QTL related to PH. It was notable that *qSW13–1* explained the hightest PV in the QTLs identified for SW. More remarkably, four novel QTLs for Oil, inculding *qOil1–1*, *qOil1–2*, *qOil10–1* and *qOil10–2* explained up to 72.73% of the PV for Oil, which suggested it might be potential loci to Oil. *qProtein1–1*, which explained 17.68% of the PV, might be an major QTL for further fine mapping. So many novel QTLs observed in the present study indicated that more germplasms need to be used for revealing the complex genetic basis of soybean.

The stability of QTL is essential for the use in a breeding programme. In the study, 31 QTLs were identified by both ICIM and CIM methods. Furthermore, one QTL for PH was identified by CIM in both experiments from 2013 and 2014. In addition, more than half of the QTLs had been reported. Five QTLs for PH detected in this study, were colocalized as reported [14, 19, 25, 26]. Two major QTLs associated with SW, *qSW13–1* and *qSW15–1*, both with the physical distance of approximate 11 Kb, explained 18.55% and 12.21 of the PV for SW, respectively. *qSW13–1* had been reported as being associated with L050–14 [27], Satt144 [28, 29] and Sat_103 [30]. *qSW15–1* had been detected in two soybean populations, derived from ‘Young’ and ‘PI416937’ (Pop1), ‘PI97100’ and ‘Coker 237’ (Pop2) [31]. Han et al. also identified the similar QTL on chr15 in the population from a cross between ‘Hefeng25’ and ‘Conrad’ [32]. Therefore, *qSW13–1* and *qSW15–1* might be considered as major and stable QTLs for further fine mapping and map-based cloning to elucidate the mechanisms of SW. In the present study, four QTLs related to Oil had been reported [28, 33–35], inculding *qOil6–1*, *qOil13–1*, *qOil19–1* and *qOil19–2*, but none of them explained more than 10% of the PV. Lee et al. [36] reported cr274_1 associated with Protein on chr15 using a population derived from ‘Young’ and ‘PI416937’. The QTL for Protein between Satt173 and Satt581 on chr10 had been identified [37], similar with the result of Liu et al. [38]. Our study detected two QTLs related to Protein, *qProtein10–1* and *qProtein15–1*, with 16.83% and 14.36% of the PVE, respectively, mapped on the same area as previous studies [36–38], might be good for MAS breeding and accurate QTL localization.

Several QTLs of various traits can map to the same locus [14, 39]. In this study, two pairs of QTLs, *q2013Oil1–1* and *q2013Protein1–1* as well as *q2014Oil10–2* and *q2013protein10–1*, with *inverse additive effect for Oil and Protein*, were located in the same marker interval (Fig. 4; Additional file 4), which implies that *q2013Oil1–1* and *q2014Oil10–2* not only control oil content in seeds but also affect protein content in seeds. It is consistent with previous reports that an negative correlation is in agreement between protein and oil concentration in soybean seeds [40, 41].

Knowledge of epistasis effect, which is defined as interactions between alleles of two or more genetic loci, is essential to understand the genetic mechanism and the gene networks underlying complex traits. In this study, 3 pairs of epistatic QTLs for Protein were identified by ICIMapping-EPI. However, these epistatic QTLs did not display additive effect alone. It might be considered modifying genes that have no significant effects alone but might affect the expression of Protein related genes through epistatic interactions. Nevertheless, epistatic interaction could not be detected in some map populations [42]. It might be the reason that no epistatic effect observed for PH, SW and Oil in the present study.

### Gene mining based on precise QTLs

As the average ratio of gene to physical distance is about 1 gene per 20 Kb in soybean genome [43], the accuracy of QTL mapping is of great benifit to gene localization and identification. The physical distance of 11 QTLs in the current study was shorter than 100 Kb, which might lead to a direct opportunity to find candidate genes by bioinformatics tools. For example, the minimum confidence interval of *qPH8–2* was 8.3 Kb, which was much shorter than 0.09 Mb detected previously [26]. Furthermore, *Glyma.08 g337400*, encoding a transducin/WD40 repeat-like superfamily protein, was predicted in the interval of *qPH8–2*, which might be a promising target to engineer transgenic plants with higher biomass and improved growth development for plant-based bioenergy production [44]. In the interval of *qPH17–1*, *Glyma.17 g169100*, encoding a 2OG-Fe(II) oxygenase superfamily protein, was one of the important gibberellin oxidase genes [45], which might affect plant height directly. *Glyma.17 g167700*, encoding a growth regulator *CYCLIN D3–2*, expressed in growing shoot apices preferentially [46–48]. In the interval of *qSW5–1*, *Glyma.05 g055700*, encoding beta vacuolar processing enzyme, was involved in seed coat formation at the early stage of seed development [49]. In the interval of *qSW12–1*, ribosomal protein L23AB encoded by *Glyma.12 g066700*, was required for normal development [50]. *Glyma.01 g106000* and *Glyma.01 g106100* in the interval of *qOil1–2*, encoding Glutathione S-transferase TAU 8, might influence Oil by suppressing lipid peroxidation [51]. There were 3 genes found in the confidence interval of *qOil10–2*, which explained 20.17% of the PV for oil, inculding *Glyma.10 g181800*, *Glyma.10 g181900* and *Glyma.10 g182000*. Moreover, *Glyma.10 g181900*, encoding a trigalactosyldiacylglycerol 1 protein (*TGD1*), affected the metabolic flux of chloroplast lipid synthesis and photosynthetic capacity, which resulted in the change of fatty acid in leaf and seed [52–55]. It was noteworthy that *qOil10–2* was placed in the same confidence intervals as *q2013protein10–1*. The inverse relationship between oil and protein in soybean seed is well documented in the previous reports [40, 41]. However, little study of *TGD1* had been reported on the function of protein accumulation in seeds. In the interval of *qProtein10–1*, *Glyma.10 g183900*, encoding peptide transporter 3, contributed to nitrogen allocation and grain Yield [56], *Glyma.10 g184900*, encoding a ureidoglycolate amidohydrolase, played a key role in nitrogen transport and storage [57–59]. In a word, on the basis of the physical position of these precise QTLs detected using a high-density map in the present study, it would be easy to find candidate gene.

## Conclusions

In this study, we genotyped a RIL population (Huapidou × Qihuang26) by SLAF-seq. A high-density genetic map for soybean was constructed and used to identify QTLs associated with four traits, including plant height, seed weight, oil content seed and protein content in seed. A total of 35 QTLs related to four traits were identified. Of these QTLs, 21 QTLs were coincident with previous research. Furthermore, three pairs epistatic QTLs involving 6 loci on 5 chromosomes were identified for Protein. In addition, 43 genes with tissue-specific expression patterns were considered to be potential genes in further study. Our findings might be of great useful for MAS breeding, and could provide detailed information for accurate QTL localization.

## Methods

### Plant material and phenotyping

F8:9 and F8:10 populations of 149 RILs derived from a cross between ‘Huapidou (ZDD09982)’ and ‘Qihuang26 (ZDD23189)’ were planted in the experiment field of Shandong Academy of Agricultural Sciences in Jinan, Shandong Province, China, in 2013 and 2014, respectively. Each individual was planted in one row using single seed sowing; each row was 3 m, with 50 cm row spacing and 10 cm plant spacing, with three replicates. Five plants in each replicate were selected randomly to calculate the plant height (PH). The weight of 100 random filled seeds was measured as seed weight (SW). Oil and protein in soybean seed were detected by DA 7200 NIR food analyzer (Perten, Switzerland). SW, Oil and Protein were repeat 3 times in each replicate. Frequency distribution, descriptive statistics, the broad-sense heritability (*h*^*2*^) and the analysis of variance for RIL population and parents were analyzed with the SPSS statistics 17.0 and Microsoft Excel 2010. The *h*^*2*^ was estimated as described by previous study [60].

### DNA extraction and genotyping

Seedlings of the F8:11 population of 149 RILs and parents were planted in 2016. Young healthy leaves from the two parents and RIL individuals were collected and genomic DNA was extracted by the CTAB method [61]. DNA was quantified with NanoDrop and by electrophoresis in 1% agarose gels with a λ DNA standard.

SLAF-seq was used to genotype a total of 151 samples (149 individuals and two parents) as described by previous study [7]. All polymorphic SLAFs were genotyped with consistency in the offspring and parental SNP loci. All SLAF markers should be filtered in quality assessment. A SLAF marker with parental homozygous, which had less than three SNPs, average depths of each sample above 3, was used as a high quality SLAF marker.

### Linkage map construction

Before map construction, SLAF marker should be filtered by linkage analysis, markers with the MLOD value > 3 were used to construct genetic linkage map. SLAF markers with high quality were located into 20 LGs. HighMap Strategy was used to order SLAF markers and correct genotyping errors [24]. All LGs should be undergone these procedures: first, markers were arranged by their locations on choromosome; second, genotyping errors or deletions were corrected by SMOOTH [62], according to the relationship between ordered markers; then MSTmap was used to order the map [63]; after that, SMOOTH was used again to corrected the new ordered genotype. High-quality map would be obtained after 4 or more cycles. Map distance was estimated using the Kosambi mapping function.

### QTL mapping

Based on the high-density genetic map, the QTLs underlying PH, SW, Oil and Protein were identified by QTL ICIMapping V3.3 software [64]. Inclusive Composite Interval Mapping (ICIM) and Composition interval mapping (CIM) methods were used to identify the QTLs. The threshold of logarithm of odds (LOD) score for evaluating the statistical significance of QTL effect was determined using 1000 permutations at the 5% level of significance. The location of a QTL was described according to its LOD peak location and thesurrounding region with 95% confidence interval [65]. As a result, intervals with a LOD value above 2.5 were detected as effective additive QTLs using ICIM-ADD method; the pairs of QTLs with a LOD value above 5.0 were considered as valid epistatic QTLs using ICIM-EPI method.

### Gene mining

According to the marker sequence, QTLs were projected from the genentic map onto Williams 82 physical map (Glyma.Wm82.a2.v1). The QTL with the PVE above 10% and the physical distance less than 1 Mb was used to screen the candidate genes. The QTL with the physical distance less than 100 Kb was also chosen to screen the candidate genes. Gene calls and annotations were retrieved using Glyma.Wm82.a2.v1 gene model from Soybase. The expression data of candidate genes in the seed development stages based on RNA-seq in previous study were obtained from soybase [66]. The expression values of candidate genes in flower, leaf, nodule, pod, root, root hair, seed, shoot apical meristem and stem were downloaded from phytozome (www.phytozome.org). Multiple array viewer (version 4.9.0) was used to construct the heat map to analyze the tissue-specific expression of candidate genes.

## Additional files

Additional file 1: Genotype list of 149 RILs. (XLSX 2691 kb)

Additional file 2: SLAF markers on the 20 linkage groups. (XLSX 129 kb)

Additional file 3: Information of SLAF markers on the genetic map. (PARTIAL 4956 kb)

Additional file 4: Additive QTLs identified by CIM in both experiments from 2013 and 2014. (PDF 168 kb)

## Abbreviations

chr: Chromosome
CTAB: Hexadecyl trimethyl ammonium bromide
DAF: Day after flowering
GBS: Genotyping-by-sequencing
GC: Guanine-cytosine
ICIM: Inclusive composite interval mapping
LGs: Linkage groups
LOD: Logarithm of odds
MAS: Molecular marker-assisted selection
Oil: Oil content in soybean seeds
PH: Plant height
Protein: Protein content in soybean seeds
PV: Phenotypic variance
PVE: Phenotypic variance explained
QTL: Quantitative trait locus
RAD-seq: Restriction-site associated DNA sequencing
RH: Root hair
RIL: Recombinant inbred lines
SAM: Shoot apical meristem
SLAF-seq: Specific length amplified fragment sequencing
SNP: Single nucleotide polymorphisms
SW: 100-seeds weight
