# Supplementary material for: Construction of a high-density genetic map and mapping of QTLs for soybean (Glycine max) agronomic and seed quality traits by specific length amplified fragment sequencing
Source: BMC Genomics. 2018 Aug 29;19:641. doi: 10.1186/s12864-018-5035-9 (PMC6116504; doi:10.1186/s12864-018-5035-9)
Supplement: Supplementary file 4 — Additive QTLs identified by CIM in both experiments from 2013 and 2014. (PDF 168 kb) [file 12864_2018_5035_MOESM4_ESM.pdf]

Additional file 4 Additive QTLs identified by CIM in both experiments from 2013 and 2014

QTL[a]	Chr[b]	Left marker	Right marker	Genetic position (cM)			LOD[c]	PVE(%)[d]	ADD[e]
				Start	End	Distance			
q2013PH 1-1	1	Marker4217409	Marker4345620	21.09	22.79	1.70	3.84	4.86	4.06
q2013PH6-1	6	Marker172599	Marker293115	77.50	78.32	0.82	15.11	23.81	9.13
q2013PH7-1	7	Marker5657567	Marker5853665	50.09	51.12	1.03	6.08	7.77	-5.20
q2013PH8-1	8	Marker2962002	Marker2806136	174.90	175.24	0.33	5.44	6.83	4.87
q2013PH11-1	11	Marker1488477	Marker1487478	67.44	68.26	0.81	2.57	3.16	-3.35
q2013PH19-1	19	Marker2154660	Marker1993462	33.77	35.14	1.36	12.29	17.71	7.76
q2013SW5-1	5	Marker1713283	Marker1777477	22.63	38.81	16.18	3.15	5.04	0.50
q2013SW11-1	11	Marker1480158	Marker1628838	2.55	5.27	2.72	5.66	9.79	0.72
q2013SW11-2	11	Marker1573686	Marker1603890	59.13	65.87	6.74	7.76	14.17	-0.86
q2013SW15-1	15	Marker2580154	Marker2712589	22.49	26.30	3.81	10.37	18.36	-0.96
q2013SW20-1	20	Marker809331	Marker998443	87.30	89.41	2.11	5.11	8.27	0.64
q2013Oil1-1	1	Marker4303320	Marker4156660	61.50	66.40	4.90	19.99	20.85	0.80
q2013Oil1-2	1	Marker4417270	Marker4449632	77.85	80.37	2.51	10.13	8.23	-0.50
q2013Protein1-1	1	Marker4303320	Marker4156660	61.50	66.40	4.90	9.21	15.73	-0.54
q2013Protein5-1	5	Marker1695301	Marker1847746	74.43	77.65	3.23	5.57	9.06	-0.41
q2013Protein8-1	8	Marker2994984	Marker2762387	180.82	182.40	1.59	3.58	5.48	0.33
q2013protein10-1	10	Marker1281634	Marker1235885	180.85	183.08	2.23	3.89	5.94	-0.33
q2013Protein16-1	16	Marker3275652	Marker3256316	125.74	128.32	2.58	4.54	7.11	0.36
q2014PH5-1	5	Marker1697153	Marker1930639	102.82	103.65	0.82	3.86	2.97	-6.32
q2014PH8-1	8	Marker2999607	Marker2981023	63.87	65.77	1.90	5.57	4.38	7.51
q2014PH14-1	14	Marker5297092	Marker5034298	165.28	167.73	2.45	4.46	3.52	6.72
q2014PH17-1	17	Marker403730	Marker599785	41.57	44.66	3.08	7.83	6.63	9.36
q2014PH19-1	19	Marker2342326	Marker2131949	25.06	27.49	2.44	17.52	17.60	-15.03
q2014PH19-2	19	Marker2154660	Marker1993462	33.77	35.14	1.36	25.23	28.47	19.13
q2014SW5-1	5	Marker1790368	Marker1713283	21.62	22.63	1.01	5.50	7.22	0.76
q2014SW9-1	9	Marker4666534	Marker4706727	96.89	101.89	5.00	5.65	7.47	0.78
q2014SW12-1	12	Marker5558494	Marker5592791	18.47	23.12	4.65	14.25	22.01	-1.33
q2014SW13-1	13	Marker3613921	Marker3643913	103.37	104.05	0.68	9.11	12.93	-1.02
q2014SW20-1	20	Marker846233	Marker994705	165.31	169.83	4.52	3.69	4.97	0.65
q2014Oil1-1	1	Marker4316904	Marker4222540	119.72	124.01	4.29	4.56	4.77	-0.25
q2014Oil6-1	6	Marker158010	Marker162307	64.37	65.93	1.56	5.33	5.76	-0.28
q2014Oil10-1	10	Marker1115605	Marker1053573	9.00	9.81	0.82	7.29	7.87	-0.33
q2014Oil10-2	10	Marker1281634	Marker1235885	180.85	183.08	2.23	25.27	38.11	0.72
q2014Oil19-1	19	Marker2060818	Marker2188637	3.74	8.92	5.18	10.50	12.74	-0.41
q2014Protein4-1	4	Marker6548332	Marker6730760	31.65	32.83	1.18	8.74	10.87	0.65
q2014Protein10-1	10	Marker1235885	Marker1249450	183.08	184.24	1.16	13.99	18.93	-0.87
q2014Protein15-1	15	Marker2413618	Marker2651368	8.34	11.38	3.04	14.96	20.98	-0.90
q2014Protein17-1	17	Marker570357	Marker647757	153.81	156.82	3.01	4.60	5.23	0.46
q2014Protein18-1	18	Marker3780954	Marker3787603	97.60	98.28	0.67	4.62	5.27	0.45

[a] QTLs stable across both years were in bold; [b] chr, chromosome; [c] LOD, logarithm of odds; [d] PVE, phenotypic variance explained; [e] ADD, additive effect.
